# Skipping as an Exercise

**Published:** 1904-04-02

**Authors:** 


### The Hospital.

A Journal of

The Medical Sciences and Hospital Administration.

Vol. XXXVI.—No. 914. APRIL 2, 1904.

### SKIPPING AS AN EXERCISE.

Dr. Bond, of Gloucester, who is favourably known both to the profession and to the public on account of his exertions in the cause of vaccination, is also the main support of a society for the promotion of sanitary teaching in the city in which he resides. In this capacity he has lately put forth a pamphlet on the advantages to be derived from "skipping" as a systematic exercise; a pamphlet the first mention of which is perhaps likely to provoke a smile, but which deserves, nevertheless, to be seriously considered. Dr. Bond does not use the word to skip in its wide Johnsonian meaning, nor does he refer at all to the exercise attributed to the high hills by the Psalmist; but he intends to express only the methods of springing over a revolving rope which found much favour with the young people of fifty years ago, although, if we are not mistaken, they have fallen into at least comparative disuse in the present day. He has even gone so far as to invent an improved rope with which the exercise may be most advantageously performed, and has bestowed upon his invention the name of "Girbola," a somewhat questionable result of an alliance between γυρὸς and βολή. The merits or demerits of a name, however, cannot seriously affect those of the thing signified; and there can be no doubt that the contrivance, which consists of a rope of easily adjustable length, somewhat weighted in the centre, and protected there by being enclosed within a tube of vulcanised india-rubber, is calculated materially to facilitate the efforts of an adult who tries for the first time to recover some portion of the agility of childhood. Dr. Bond points out the wide range of muscular action which the exercise requires, the muscles of the arms, legs, and chest being called into simultaneous activity; and he dwells also upon the ease with which the required efforts can be graduated to meet the necessities of the skipper, and to keep pace with progressive improvements in his or her condition. The exercise may at first be performed almost languidly, or at the slowest pace which will allow the rope (we beg pardon, the girbola) to be kept in continuous motion, or it may be increased in speed until the feats of double or even treble skipping are accomplished, in which the girbola makes two or even three revolutions for every jump. It may be laid aside after two skips, or after twenty, or after two hundred, according to the nature of the case and the increasing strength of the performer; and it may be taken up anywhere and at any time, either out of doors or in any room or passage where a few feet of unencumbered space can be obtained. Dr. Bond strongly recommends it to be practised for a few minutes on first rising from bed, or after a bath, and speaks from personal experience of its efficacy in giving vigour to the muscles and tone to the circulation, even in a subject no longer in the heyday of youth. He specially recommends it in all the cases of weak heart in which exercises are calculated to be useful, and thinks it much better adapted to their requirements than any of the "resisted movements" which have been advised in such cases, and which can only be practised with the aid of a person trained in their performance. He also recommends it as a daily custom for all persons engaged in sedentary occupations, and especially in occupations requiring brainwork, as the best possible means of counteracting the tendency to local congestions which such occupations may involve.

There is always something contagious in enthusiasm, and we confess that Dr. Bond seems to us to have gone a long way towards proving his case, or, at all events, to have shown that a neglected child's sport may be so used as to be eminently useful to children of a larger growth. We have no recollection of a definite therapeutic position having been assigned to skipping by an earlier writer, except in the case of children suffering from what was known to the last generation as "strumous ophthalmia," a condition in which great intolerance of light often continued for many days or even weeks, and in which skipping was recommended as a vigorous exercise capable of being practised in a dark or very dimly lighted room. Ophthalmia of the kind described has disappeared before modern improvements in the direction of local treatment; but, at the same time, the attention alike of the profession and of the public has been more and more directed towards the value of systematic exercises; and a variety of contrivances for facilitating the performance of them are recommended by a proportionate number of inventors or patentees. It may be said of most of them that the exercises accomplished by their use are more or less partial, calculated to call only certain groups of muscles into action, and requiring to be both varied and prolonged before the muscular system as a whole is compelled to take its full part in the production of the successive movements. From this reproach skipping is certainly free; and the chief variation of which it admits, or which it requires, that of making the rope revolve sometimes backwards and sometimes forwards, is accomplished practically without interruption of the exercise. We cannot but feel that the chief impediment to the general acceptance of Dr. Bond's views, and the general practice of his recommendations, is to be found in their very simplicity; and we think he has done well to bring out his "girbola," even if only as a means of reaching the somewhat dull imagination of the average man. To skip with a rope would be codemned as childish by many who would regard the girbola as an important means towards the attainment of an equally important end. For our own part, we are on the side of Naaman's servants, and would urge upon our masters the public not to disregard the advice of the prophet merely on account of its freedom from theatrical display. The injunction to "skip and be well" is fairly comparable with that to "wash and be clean," and deserves at least a corresponding degree of attention.

